# Airway inflammation and asthma control in patients using salbutamol or budesonide/formoterol reliever: a randomised, controlled study

**DOI:** 10.1183/23120541.00116-2026

**Published:** 2026-09-28

**Authors:** Matthew J. Martin, Ian D. Pavord, James D. Chalmers, Dinesh Saralaya, Kay G. Roy, Manish Patel, Graham W. Clarke, Laura Belton, Annika Rutgersson, Per Gustafson, Vieshal Raja Gopal, Tim Harrison

**Affiliations:** 1 Nottingham Respiratory Research Unit, University of Nottingham, Nottingham, UK; 2 Respiratory Medicine Unit and Oxford Respiratory NIHR BRC, Nuffield Department of Medicine, University of Oxford, Oxford, UK; 3 Division of Respiratory Medicine and Gastroenterology, University of Dundee, Dundee, UK; 4 Bradford Teaching Hospitals NHS Foundation Trust and Bradford Institute for Health Research, Bradford, UK; 5 Respiratory Medicine, University College London Hospitals NHS Foundation Trust, London, UK; 6 Respiratory Department, University Hospital Wishaw, Wishaw, UK; 7 BioPharmaceuticals Research and Early Development Respiratory & Immunology, Translational Science & Experimental Medicine, AstraZeneca, Gothenburg, Sweden; 8 School of Immunobiology, King's College London, London, UK; 9 Biostatistics, BioPharmaceuticals Medical, AstraZeneca, Cambridge, UK; 10 Evinova, Gothenburg, Sweden; 11 Alexion Pharmaceuticals, Barcelona, Spain; 12 Respiratory Global Medical Affairs, BioPharmaceuticals Medical, AstraZeneca, Cambridge, UK; 13 Faculty of Medicine and Health Sciences, University of Nottingham, Nottingham, UK

## Abstract

**Background:**

Maintenance and reliever therapy (MART) with budesonide/formoterol (BUD/FORM) may offer asthma control with a single inhaler. This 6-month exploratory study monitored asthma symptoms, reliever use, lung function, airway inflammation and exacerbation events to determine treatment effectiveness in patients receiving BUD/FORM MART or BUD/FORM maintenance therapy plus short-acting β_2_-agonist (SABA).

**Methods:**

In the open-label STIFLE study (NCT03924635), patients were randomised to BUD/FORM MART (100 μg/6 μg or 200 μg/6 μg per inhalation, *pro re nata* (PRN)) or the same dose of BUD/FORM BID as maintenance therapy plus SABA (salbutamol) reliever PRN (100 μg per inhalation). Data were captured at home each day using devices connected to a smartphone *via* the STIFLE application. Occurrence of pre-defined exacerbation events required patients to attend hospital for assessment.

**Results:**

42 patients were randomised. Adherence to daily measurements exceeded 80% in both treatment groups. The proportion of patients experiencing an exacerbation was 27.8% in the BUD/FORM MART *versus* 70.8% in the BUD/FORM maintenance plus SABA group. A similar trend was observed in patients who experienced ≥1 day with ≥6 reliever uses (3 (16.7%) *versus* 9 (37.5%) patients, respectively). Patients treated with BUD/FORM MART showed less variation in airway inflammation, asthma symptom scores, lung function parameters and reliever use, *versus* BUD/FORM maintenance therapy plus SABA. No unexpected safety findings were observed.

**Conclusions:**

Patients with moderate asthma treated with BUD/FORM MART recorded less variation in asthma outcomes and lower rates of exacerbation events than those treated with BUD/FORM maintenance therapy plus SABA, suggesting improved asthma control.

## Shareable abstract

**Patients with moderate asthma receiving budesonide/formoterol (BUD/FORM) maintenance and reliever therapy had less variation in asthma outcomes and lower exacerbation rates *versus* those receiving BUD/FORM maintenance therapy plus short-acting β_2_-agonist.** https://bit.ly/48bZ2ty

## Introduction

Asthma is a chronic inflammatory disease of the airways that affects over 260 million people worldwide [1, 2]. Despite marked decline in global prevalence rates over the past 30 years, asthma is still associated with significant rates of mortality [2, 3].

Increased airway inflammation is associated with worsening of asthma symptoms and higher rates of asthma exacerbations [4]. While short-acting β_2_-agonist (SABA)-only inhalers can provide symptomatic relief, they do not address the underlying inflammatory cause of asthma and, as a result, do not protect against exacerbations [5–7]. Over-reliance on SABA inhalers can be associated with increased exacerbations and mortality risk, and SABA over-use is recognised as a marker of poor asthma control [8, 9]. Nearly 40% of people using SABAs at the time of their asthma-related death had been prescribed more than 12 SABAs in the year before they died [9]. In the UK, it is estimated that 38% of people with asthma over-use their SABA-only inhalers [5].

Combinations of inhaled corticosteroid/long-acting β_2_-agonists (ICS/LABAs), such as budesonide/formoterol (BUD/FORM), treat asthma symptoms as well as address underlying inflammation, thereby reducing the risk of exacerbations [10]. Maintenance and reliever therapy (MART) with BUD/FORM offers the possibility of controlling asthma with a single inhaler [11]. Compared with separate ICS/LABA and SABA regimens, use of BUD/FORM MART has been shown to reduce the number of *pro re nata* (PRN) inhalations for symptom relief while reducing severe exacerbations [11, 12].

This exploratory study examined airway inflammation (measured as fractional exhaled nitric oxide (*F*_ENO_)) [13], asthma symptoms, lung function and reliever use, assessed daily over a period of 6 months, and surrounding exacerbation events, in adults with asthma receiving BUD/FORM MART or BUD/FORM maintenance therapy plus SABA. The primary objective was to descriptively characterise the relationship between inflammation, asthma symptoms, lung function and reliever use measured daily over 24 weeks of treatment in each treatment arm. The secondary objective was to similarly profile symptoms and characteristics surrounding any recorded exacerbation event.

## Materials and methods

### Study design

STIFLE (NCT03924635) was a phase 4, randomised, exploratory, open-label study. All patients received BUD/FORM maintenance therapy (100 μg/6 μg or 200 μg/6 μg per inhalation, twice daily based on their ICS dose at study entry) and SABA reliever (100 μg per inhalation, PRN) during the 2-week period before randomisation. Patients were then randomised 1:1, with randomisation stratified according to their ongoing ICS dose at study entry (low or medium), to BUD/FORM MART (100 μg/6 μg or 200 μg/6 μg per inhalation based on ICS dose, twice daily for maintenance and PRN for relief) or the same dose of BUD/FORM BID as maintenance therapy plus salbutamol (SABA) reliever (100 μg per inhalation, PRN) for 24 weeks (figure 1a).

**FIGURE 1:**
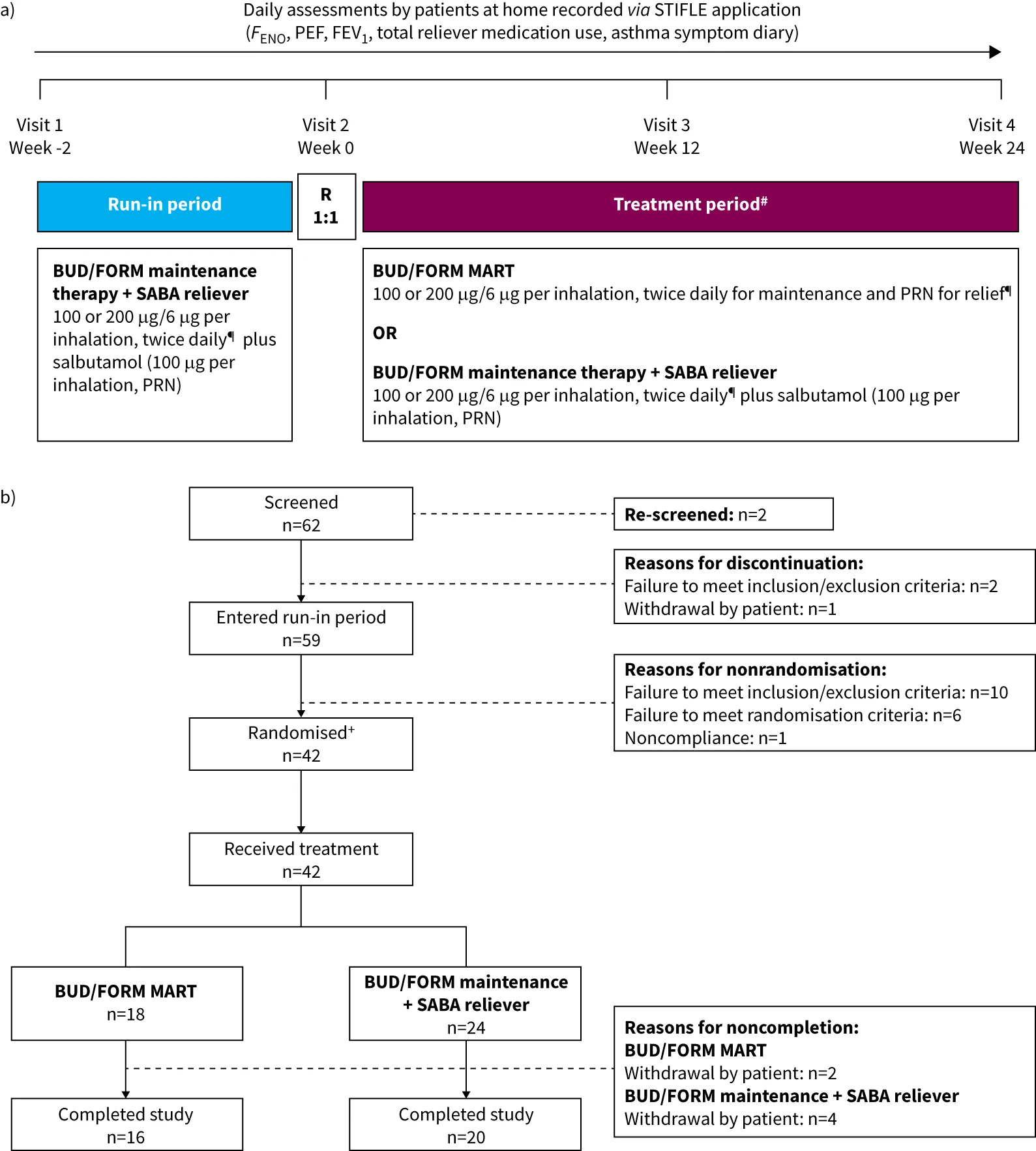
a) Study design and b) patient disposition. ^#^: Patients experiencing an exacerbation event of interest were instructed to attend a hospital visit at the earliest possible date and were required to attend three further visits at approximately 4-day intervals. ^¶^: Based on inhaled corticosteroid dose at study entry. ^+^: All randomised patients were included in the analyses. BUD: budesonide; *F*_ENO_: fractional exhaled nitric oxide; FEV_1_: forced expiratory volume in 1 s; FORM: formoterol; MART: maintenance and reliever therapy; PEF: peak expiratory flow; PRN: *pro re nata*; R: randomisation; SABA: short-acting β_2_-agonist.

Throughout the study, patients were required to capture data at home each day using four devices connected to a smartphone *via* the STIFLE application (figure 1a). *F*_ENO_ was measured in the morning using the Vivatmo Me (Bosch) device. Peak expiratory flow (PEF) and forced expiratory volume in 1 s (FEV_1_) were captured in the morning and evening with the Spirobank Smart (MIR) device. For the measurement of reliever use, the Turbu+ (SmartTurbo) and the Hailie (Adherium) devices recorded use of BUD/FORM and salbutamol, respectively. Patients recorded asthma symptom score and reliever use twice daily (morning and evening) in an asthma symptom diary within the STIFLE application. Patients were asked to record reliever use because device-measured reliever use may have been overestimated since SABA inhalers must be primed when new or if they have not been used for some time. Asthma symptoms were scored twice daily on a scale of 0–3 (morning and evening scores combined), with 0 representing no asthma symptoms and 3 representing an inability to perform normal activities or sleep due to asthma symptoms. All measurements were made before taking study medications.

Three pre-defined exacerbation events of interest required patients to attend a hospital visit at the earliest possible date, as follows: 1) severe exacerbations (SevEx) (events treated with at least 3 days of oral corticosteroids, an emergency room visit (or other urgent care visit) resulting in the administration of systemic corticosteroids or an inpatient hospitalisation for asthma), 2) a composite end-point for exacerbations in asthma event (CompEx) (a SevEx or diary-based event, identified using an established algorithm as described in the study protocol, involving worsening PEF, increased reliever use or worsening symptoms) [14], and 3) single days (24 h) with at least six reliever uses.

Serious adverse events (SAEs) and adverse events (AEs) leading to treatment discontinuation were reported by the patient at study visits.

### Study participants

Patients (at least 18 years of age) were recruited from six sites within the UK. All patients had been diagnosed with moderate asthma for at least 6 months, were receiving step 3 or step 4 (ICS/LABA, low- or medium-dose for asthma, in accordance with Global Initiative for Asthma (GINA) 2018 guidelines [15]) treatment for at least 3 months and had experienced at least one episode of symptom worsening requiring reliever over-use (defined as more than the standard for the individual patient) in the last 30 days. Patients had to be able to perform at home *F*_ENO_ and spirometry assessments and to complete an asthma symptom diary on a daily basis.

Current smokers, or people with any significant disease or a drug/substance abuse disorder that could interfere with the conduct of the study, were excluded from participation. Additional exclusion criteria included any asthma worsening requiring a change in asthma treatment and acute upper or lower respiratory tract infections requiring treatment within 30 days before the first study visit. People with a history of life-threatening asthma requiring intubation, a medical condition that could influence *F*_ENO_, concurrent respiratory disease or a severe asthma exacerbation within 30 days of screening were also not eligible to participate.

The study was performed in accordance with the Declaration of Helsinki and with the International Council for Harmonisation/Good Clinical Practice guidelines, as well as in compliance with applicable regulatory requirements. An Independent Ethics Committee (Health and Social Care Research Ethics Committee, Lisburn, Northern Ireland, UK) reviewed and approved the study protocol. All participants provided written informed consent before study participation.

### Statistical analyses

This study was exploratory in nature with no formal hypotheses and was not designed or powered to test for significant differences between treatment arms. All analyses were descriptive and conducted using SAS Version 9.3. Analyses were based on the full analysis set, which comprised all randomised patients with at least one post-baseline measurement, irrespective of protocol adherence and continued study participation. Outcomes were assessed between treatment arms and at the individual patient-level. Safety outcomes were assessed in the safety analysis set, which included all patients who received at least one dose of study medication.

Mean overall adherence by patients to daily measures during the treatment period was calculated as:

$$\frac{\mathrm{Number}\;\mathrm{of}\;\mathrm{actual}\;\mathrm{sessions}\;\mathrm{with}\;\mathrm{complete}\;\mathrm{data}}{\mathrm{Number}\;\mathrm{of}\;\mathrm{expected}\;\mathrm{sessions}\;\mathrm{with}\;\mathrm{complete}\;\mathrm{data}}\times 100$$

The study planned to randomise 60–80 patients, with the goal of having at least 54 patients completing the study, which was considered feasible to allow for individual patient plots to be generated and reviewed as per the primary objective of the study. Recruitment was put on hold during the COVID-19 pandemic (March–November 2020). Afterwards, three additional sites were included to aid recruitment. However, the study was stopped early due to continuing recruitment issues attributed to a reduction in exacerbation events observed in clinical practice, resulting in a smaller sample size than originally planned.

## Results

### Patient characteristics

A total of 42 patients (64.3% female, mean±sd age 49.2±15.8 years) were randomised to receive treatment with BUD/FORM MART (n=18) or BUD/FORM as maintenance therapy plus SABA reliever (n=24) (figure 1b) (the first participant was enrolled on 1 August 2019 and the last participant visit was on 16 December 2022). Mean follow-up time was 159.9 days for those receiving BUD/FORM MART and 156.2 days for those receiving BUD/FORM as maintenance therapy plus SABA. Additionally, two and four patients in the respective treatment arms withdrew from the study early (figure 1b).

Baseline demographic and clinical characteristics were generally comparable between the treatment arms (table 1), although there were a few notable differences. Patients treated with BUD/FORM MART were older (mean age: 54.1 *versus* 45.5 years), included more former smokers (five *versus* two) and had a lower average morning and evening PEF (morning mean: 374.1 *versus* 426.0 L·min^−1^; evening mean: 392.0 *versus* 440.6 L·min^−1^) compared with those receiving BUD/FORM as maintenance therapy plus SABA.

**TABLE 1:** Baseline demographic and clinical characteristics of patients receiving budesonide (BUD)/formoterol (FORM) maintenance and reliever therapy (MART) or BUD/FORM maintenance therapy plus short-acting β2-agonist (SABA) (full analysis set)

Parameter	BUD/FORM MART (n=18)	BUD/FORM maintenance+SABA (n=24)	Total (n=42)
**Age, years**	54.1±14.6	45.5±16.0	49.2±15.8
**Sex, female**	12 (66.7)	15 (62.5)	27 (64.3)
**Race**			
White	17 (94.4)	22 (91.7)	39 (92.9)
Asian	0	1 (4.2)	1 (2.4)
Other	1 (5.6)	1 (4.2)	2 (4.8)
**BMI, kg·m^−2^**	33.3±8.5	31.7±9.0	32.4±8.7
**Smoking status**			
Non-smoker	13 (72.2)	22 (91.7)	35 (83.3)
Former smoker	5 (27.8)	2 (8.3)	7 (16.7)
**Time since smoking cessation, years**	24.0±15.7	38.5±9.2	28.1±15.1
**Smoking consumption, pack-years**	7.0±4.1	2.5±2.1	5.7±4.1
**Asthma duration, years**	21.4±14.1	25.9±13.6	24.0±13.8
**Prior use of ICS** ^#^			
Low dose	3 (16.7)	7 (29.2)	10 (23.8)
Medium dose	12 (66.7)	17 (70.8)	29 (69.0)
Missing	3 (16.7)	0	3 (7.1)
**Number of exacerbations treated with systemic CS in the last 12 months**	1.3±1.1	1.3±1.2	1.3±1.2
**Number of exacerbations leading to ER visit and treatment with systemic CS experienced in last 12 months**	0.4±0.8	0.2±0.4	0.3±0.6
**Number of exacerbations leading to hospitalisation and treatment with systemic CS experienced in last 12 months**	0.2±0.5	0.3±0.6	0.2±0.6
**Time since most recent exacerbation, days**	200.5±115.1	178.8±97.5	187.7±104.0
**Baseline defined as mean values over last 10 days of run-in period prior to randomisation, mean±sd (median (Q1, Q3))**			
**Average total asthma symptom score**	1.5±0.8 (1.7 (0.8, 2.0))	1.5±0.7 (1.5 (0.9, 2.0))	1.5±0.8 (1.5 (0.9, 2.0))
**Average total reliever medication use, number of occasions**	0.8±0.7 (0.6 (0.3, 1.2))	1.0±1.0 (0.7 (0.4, 1.2))	0.9±0.9 (0.7 (0.3, 1.2))
**Night-time awakening days**	18.0±19.9 (10.6 (0.0, 30.0))	14.7±27.0 (10.0 (0.0, 10.6))	16.1±24.0 (10.0 (0.0, 20.0))
**Asthma control days**	22.2±28.8 (5.0 (0.0, 40.0))	19.6±21.6 (15.0 (0.0, 30.0))	20.7±24.6 (10.0 (0.0, 30.0))
**Reliever-free days**	55.0±19.8 (50.0 (40.0, 70.0))	49.6±17.6 (50.0 (45.0, 60.0))	51.9±18.5 (50.0 (40.0, 60.0))
**Symptom-free days**	22.2±28.8 (5.0 (0.0, 40.0))	19.6±21.6 (15.0 (0.0, 30.0))	20.7±24.6 (10.0 (0.0, 30.0))
**Average *F*_ENO_, ppb**	29.6±29.0 (17.8 (10.5, 32.6))	21.9±13.1 (17.9 (12.1, 26.7))	25.2±21.5 (17.9 (11.4, 30.7))
**Average morning PEF, L·min^−1^**	374.1±99.0 (356.5 (301.4, 423.5))	426.0±116.6 (396.7 (350.1, 511.5))	403.8±111.2 (378.6 (332.5, 448.1))
**Average evening PEF, L·min^−1^**	392.0±94.6 (379.1 (324.5, 424.9))	440.6±107.4 (418.8 (369.4, 508.2))	419.8±103.8 (398.4 (355.8, 461.7))
**Average morning FEV_1_, L**	2.1±0.7 (1.9 (1.6, 2.9))	2.5±0.8 (2.3 (1.9, 2.9))	2.3±0.7 (2.2 (1.8, 2.9))
**Average evening FEV_1_, L**	2.2±0.6 (2.0 (1.6, 2.7))	2.5±0.7 (2.3 (2.0, 2.9))	2.3±0.7 (2.2 (1.8, 2.8))

Data are mean±sd or n (%), unless otherwise stated. Total asthma symptoms score was the sum of non-missing morning and evening scores; higher scores represent more severe symptoms. Total reliever medication use was the sum of non-missing morning and evening reliever medication use. BMI: body mass index; CS: corticosteroid; ER: emergency room; *F*_ENO_: fractional exhaled nitric oxide; FEV1: forced expiratory volume in 1 s; ICS: inhaled corticosteroid; PEF: peak expiratory flow; Q1: first quartile; Q3: third quartile. ^#^: Based on [15].

### Exacerbation events

Overall, the number of patients with any exacerbation event of interest was 5 (27.8%) in the BUD/FORM MART group and 17 (70.8%) in the BUD/FORM maintenance plus SABA group. Fewer patients in the BUD/FORM MART arm than the BUD/FORM maintenance plus SABA arm experienced ≥1 SevEx or CompEx event (2 (11.1%) *versus* 7 (29.2%) and 4 (22.2%) *versus* 12 patients (50.0%), respectively). Similarly, fewer patients in the BUD/FORM MART arm experienced ≥1 day with ≥6 reliever uses (3 (16.7%) *versus* 9 patients (37.5%)). Of the total CompEx events reported, there was a similar trend in the proportion of SevEx events within each arm (2/8 events (25.0%) *versus* 8/15 events (53.3%), respectively).

### Asthma-related outcomes captured with the STIFLE application

Median overall adherence by patients to daily measurements post-randomisation for the BUD/FORM MART treatment arm ranged from 84.8% (evening FEV_1_) to 92.3% (*F*_ENO_). Adherence rates were broadly similar for the BUD/FORM maintenance therapy plus SABA arm, ranging from 86.4% (evening PEF) to 90.0% (morning asthma symptom diary). Across treatment arms, individual patient adherence to daily measurements post-randomisation ranged from a minimum of 22.6% to a maximum of 99.4%.

Over 24 weeks, patient-recorded *F*_ENO_, total asthma symptom score, PEF and FEV_1_ outcomes, and total reliever medication use was variable in both treatment arms, with a trend towards less variation in the BUD/FORM MART arm (figure 2 and supplementary figure S1). Across outcomes, the median of individual patient standard deviations trended lower, and interquartile ranges as an indicator of inter-group variability were generally narrower in the BUD/FORM MART treatment arm compared with the BUD/FORM maintenance plus SABA arm (figure 2 and supplementary figure S1). Importantly, patient-level data showed pronounced variability for all parameters, both between patients and within individual patients (figure 3 and supplementary figure S2). Notably, patients with exacerbations did not necessarily have poorer asthma outcomes; for instance, some patients had low *F*_ENO_ values on average whereas others had high *F*_ENO_ values on average (figures 2a and 3a).

**FIGURE 2:**
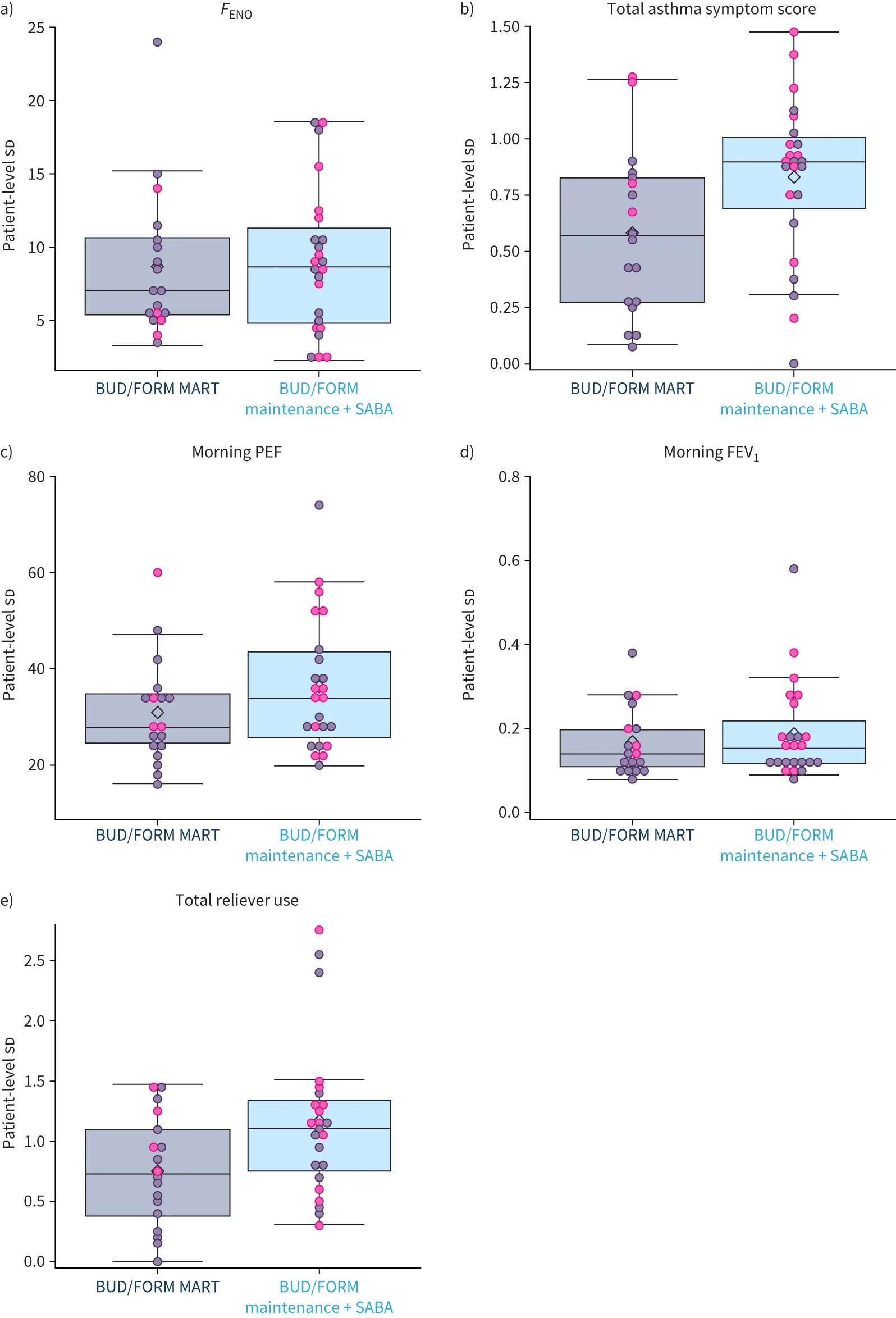
Variation in a) fractional exhaled nitric oxide (*F*_ENO_), b) total asthma symptom score, c) morning peak expiratory flow (PEF), d) morning forced expiratory volume in 1 s (FEV_1_) and e) total reliever medication use in patients receiving budesonide (BUD)/formoterol (FORM) maintenance and reliever therapy (MART) or BUD/FORM maintenance therapy plus short-acting β2-agonist (SABA) during the 24-week treatment period (full analysis set^#^). Dots show sd values for a given parameter during the 24-week treatment period for individual patients; pink dots represent patients who experienced a clinical event (≥1 severe exacerbation (SevEx) and/or composite surrogate end-point for severe exacerbations of asthma (CompEx)) and purple dots represent patients without a clinical event (without ≥1 SevEx and/or CompEx). Box shows the interquartile range (IQR), spanning from the first to third quartile, representing the middle 50% of the data. The horizontal line inside the box represents the median. Whiskers extending from the box represent the minimum and maximum nonoutlier values (smallest and largest values within 1.5×IQR from the quartiles). Individual points plotted outside of the whiskers are “outliers” (values that are more than 1.5×IQR from the quartiles). The diamond inside the box represents the mean. ^#^: All randomised patients with ≥1 post-baseline measurement. Summary statistics are based on individual patient-level sd to summarise variation over the observational period by treatment arm.

**FIGURE 3:**
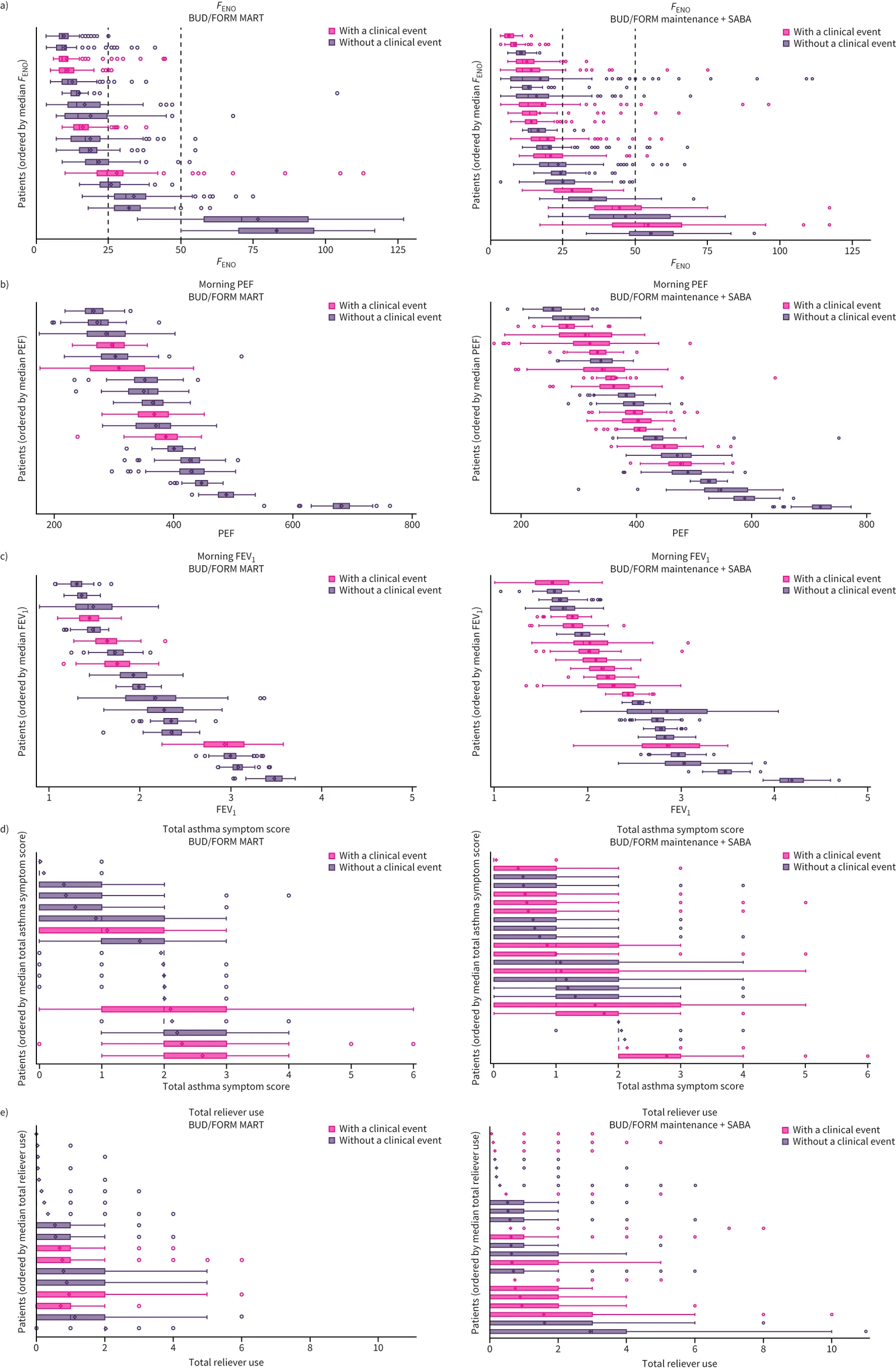
Patient-level variation in a) fractional exhaled nitric oxide (*F*_ENO_), b) morning peak expiratory flow (PEF), c) morning forced expiratory volume in 1 s (FEV_1_), score d) total asthma symptom score and e) total reliever medication use in adults with asthma receiving budesonide (BUD)/formoterol (FORM) maintenance and reliever therapy (MART) or BUD/FORM maintenance therapy plus short-acting β2-agonist (SABA) during the 24-week treatment period (full analysis set). Each line represents an individual patient; pink denotes those with a clinical event (≥1 severe exacerbation (SevEx) and/or composite surrogate end-point for severe exacerbations of asthma (CompEx)); purple denotes those without a clinical event (without ≥1 SevEx and/or CompEx). Box shows the interquartile range (IQR), spanning from the first to third quartile, representing the middle 50% of the data. The horizontal line inside the box that marks the median. Whiskers extending from the box represent the minimum and maximum nonoutlier values (smallest and largest values within 1.5×IQR from the quartiles). Individual points plotted outside of the whiskers are “outliers” (values that are more than 1.5×IQR from the quartiles). The diamond inside the box represents the mean.

### Safety outcomes

No new or unexpected safety findings were observed (table 2). No AEs were reported in the BUD/FORM MART arm. One SAE of central nervous system infection was reported in one patient in the BUD/FORM maintenance plus SABA arm. No AEs with an outcome of death or AEs leading to treatment discontinuation were reported.

**TABLE 2:** Number and duration of exacerbation events of interest in patients receiving budesonide (BUD)/formoterol (FORM) maintenance and reliever therapy (MART) or BUD/FORM maintenance therapy plus short-acting β2-agonist (SABA) during the 24-week treatment period (full analysis set)

Event	BUD/FORM MART (n=18)	BUD/FORM maintenance+SABA (n=24)
**All exacerbation events of interest**		
Patients with ≥1 event, n (%)	5 (27.8)	17 (70.8)
Total number of events	13	41
**SevEx events**		
Patients with ≥1 exacerbation, n (%)	2 (11.1)	7 (29.2)
Total number of exacerbations	2	8
Duration, days (mean±sd)	7.5±0.7	11.6±6.9
**CompEx events**		
Patients with ≥1 exacerbation, n (%)	4 (22.2)	12 (50.0)
Total number of exacerbations	8	15
Duration, days (mean±sd)	4.0±2.4	7.9±6.8
**Single day with ≥6 reliever uses**		
Patients with ≥1 event, n (%)	3 (16.7)	9 (37.5)
Total number of events	3	18
Duration, days (mean±sd)	1.0±0.0	3.7±4.0

CompEx: composite surrogate end-point for severe exacerbations of asthma; SevEx: severe exacerbation.

## Discussion

The aim of this exploratory study was to generate real-world insights on asthma control and exacerbation risk, using daily digital home monitoring, in adults with moderate asthma treated with either BUD/FORM MART or BUD/FORM maintenance therapy plus SABA reliever [10]. Over 24-weeks of self-monitoring, patients in the BUD/FORM MART treatment arm experienced fewer exacerbation events of interest and single days with ≥6 reliever uses relative to those in the BUD/FORM maintenance therapy plus SABA arm. Our finding of fewer SevEx events in the BUD/FORM MART group *versus* the BUD/FORM maintenance plus SABA group is consistent with that of a previous study [12]. The observation that patients receiving BUD/FORM MART reported fewer CompEx events with SevEx events, compared with those receiving BUD/FORM as maintenance therapy with SABA, could be due to the delivery of additional ICS at the point of symptom worsening [10].

Patients receiving BUD/FORM MART had less variation in airway inflammation and lung function and reported less variation in asthma symptoms than those treated with BUD/FORM maintenance therapy plus SABA. Reduced variability in these parameters is consistent with the mechanism of action of BUD/FORM MART, which is designed for the rapid relief of asthma symptoms and the reduction of exacerbation risk [11, 12]. However, as all patients were receiving BUD/FORM as maintenance treatment, this consistency would have controlled inflammation to some degree and may account for the limited variability in *F*_ENO_ observed herein. Additionally, adherence with maintenance treatment is usually better in clinical trials than clinical practice; therefore, reported variability may have been less than is typically seen in everyday clinical practice. The lower variability in total reliever use observed in patients who received BUD/FORM MART may reflect the longer duration of bronchodilation associated with its use as a reliever medication, potentially due to a stabilising effect on airway smooth muscle [10] or due to additional ICS use in response to symptoms.

The STIFLE application is a novel tool that allows for the capture of individual patient-level data in a home-based setting [10]. By gathering patient data on a day-to-day basis, rather than only during periods of symptom deterioration or exacerbation, this analysis provides insight into key parameter changes over a continuous 24-week period. However, several different operational and data synchronisation issues contributed to missing data; and missing data were not imputed. The following three general patterns were identified regarding patients with missing daily measurements: 1) patients performing assessments regularly but not necessarily daily; 2) patients missing daily assessments for multiple consecutive days, typically resulting in large gaps in data availability for all measures; and 3) patients withdrawing consent and not completing the study.

An imbalance in the number of patients between treatment arms was observed despite randomisation being blocked. The smaller number of patients in the BUD/FORM MART treatment arm may have influenced the trending differences observed between treatment arms. The imbalance in randomisation was likely due to recruitment ending early (due to the low number of acute exacerbations observed) and the increased number of study sites, particularly where only small numbers of patients were enrolled. One potential reason for low recruitment is the perception among patients that daily assessments, and the training required from nurses to take these measurements, are burdensome. However, adherence rates were high among randomised patients, ranging from a median of 84.8 to 92.3% across outcomes and treatment arms. Another limitation of the study is the generalisability of the results, which may be affected by the small sample size, the COVID-19 pandemic and the fact that all patients were from the UK. It should also be noted that some patients completed the study early, which may have affected variability estimates for these individuals compared with those who completed the full follow-up period.

### Conclusions

Over a 24-week monitoring period, patient and health device recorded measures of airway inflammation, asthma symptoms, lung function and reliever medication use tended to be less variable in patients with moderate asthma taking BUD/FORM MART when compared to those taking BUD/FORM maintenance plus SABA. Lower rates of exacerbation events of interest were observed in the BUD/FORM MART group. These findings suggest improved asthma control in the BUD/FORM MART group and are consistent with the application of BUD/FORM as an anti-inflammatory reliever. These data support the GINA treatment preference for ICS-containing reliever medication over SABA reliever medication in asthma management [16].

## Data Availability

Data underlying the findings described in this manuscript may be obtained in accordance with AstraZeneca's data sharing policy described at https://astrazenecagrouptrials.pharmacm.com/ST/Submission/Disclosure. Data for studies directly listed on Vivli can be requested through Vivli at www.vivli.org. Data for studies not listed on Vivli could be requested through Vivli at https://vivli.org/members/enquiries-about-studies-not-listed-on-the-vivli-platform/. The AstraZeneca Vivli member page is also available outlining further details: https://vivli.org/ourmember/astrazeneca/.

## Supplementary material

**Please note:** supplementary material is not edited by the Editorial Office, and is uploaded as it has been supplied by the author. Supplementary material 00116-2026.SUPPLEMENT Figure S1 00116-2026.SUPPLEMENT1 Figure S2 00116-2026.SUPPLEMENT2

## References

[1] World Health Organization. Asthma. Date last updated: 28 April 2026. Date last accessed: 9 July 2026. www.who.int/news-room/fact-sheets/detail/asthma

[2] Wang Z, Li Y, Gao Y, et al. Global, regional, and national burden of asthma and its attributable risk factors from 1990 to 2019: a systematic analysis for the Global Burden of Disease Study 2019. Respir Res 2023; 24: 169. doi:10.1186/s12931-023-02475-6 PMC10288698 37353829

[3] Yuan L, Tao J, Wang J, et al. Global, regional, national burden of asthma from 1990 to 2021, with projections of incidence to 2050: a systematic analysis of the global burden of disease study 2021. EClinicalMedicine 2025; 80: 103051. doi:10.1016/j.eclinm.2024.103051 PMC11764843 39867965

[4] Tillie-Leblond I, Montani D, Crestani B, et al. Relation between inflammation and symptoms in asthma. Allergy 2009; 64: 354–367. doi:10.1111/j.1398-9995.2009.01971.x 19210358

[5] Janson C, Menzies-Gow A, Nan C, et al. SABINA: an overview of short-acting β_2_-agonist use in asthma in European countries. Adv Ther 2020; 37: 1124–1135. doi:10.1007/s12325-020-01233-0 PMC7089727 31981105

[6] Kaplan A, Mitchell PD, Cave AJ, et al. Effective asthma management: is it time to let the AIR out of SABA? J Clin Med 2020; 9: 921. doi:10.3390/jcm9040921 PMC7230470 32230875

[7] Noorduyn SG, Qian C, Johnston KM, et al. SABA use as an indicator for asthma exacerbation risk: an observational cohort study (SABINA Canada). ERJ Open Res 2022; 8: 00140–2022. doi: 10.1183/23120541.00140-2022 PMC9511146 36171990

[8] Nwaru BI, Ekström M, Hasvold P, et al. Overuse of short-acting β_2_-agonists in asthma is associated with increased risk of exacerbation and mortality: a nationwide cohort study of the global SABINA programme. Eur Respir J 2020; 55: 1901872. doi:10.1183/13993003.01872-2019 PMC7160635 31949111

[9] Royal College of Physicians. Why asthma still kills. Date last updated: 18 July 2016. Date last accessed: 9 July 2026. www.rcp.ac.uk/resources/why-asthma-still-kills/

[10] Harrison T, Pavord ID, Chalmers JD, et al. Variability in airway inflammation, symptoms, lung function and reliever use in asthma: anti-inflammatory reliever hypothesis and STIFLE study design. ERJ Open Res 2020; 6: 00333–2019. doi: 10.1183/23120541.00333-2019 PMC7276524 32550224

[11] Cusack RP, Satia I, O'Byrne PM. Asthma maintenance and reliever therapy: should this be the standard of care? Ann Allergy Asthma Immunol 2020; 125: 150–155. doi:10.1016/j.anai.2020.04.009 32339657

[12] Buhl R, Kuna P, Peters MJ, et al. The effect of budesonide/formoterol maintenance and reliever therapy on the risk of severe asthma exacerbations following episodes of high reliever use: an exploratory analysis of two randomised, controlled studies with comparisons to standard therapy. Respir Res 2012; 13: 59. doi:10.1186/1465-9921-13-59 PMC3561645 22816878

[13] Taylor DR, Pijnenburg MW, Smith AD, et al. Exhaled nitric oxide measurements: clinical application and interpretation. Thorax 2006; 61: 817–827. doi:10.1136/thx.2005.056093 PMC2117092 16936238

[14] Fuhlbrigge AL, Bengtsson T, Peterson S, et al. A novel endpoint for exacerbations in asthma to accelerate clinical development: a *post-hoc* analysis of randomised controlled trials. Lancet Respir Med 2017; 5: 577–590. doi:10.1016/S2213-2600(17)30218-7 28583396

[15] Global Initiative for Asthma. Global strategy for asthma management and prevention. Date last updated: 2018. Date last accessed: 9 July 2026. https://ginasthma.org/wp-content/uploads/2018/04/wms-GINA-2018-report-V1.3-002.pdf

[16] Global Initiative for Asthma. Global strategy for asthma management and prevention. Date last updated: 2024. Date last accessed: 9 July 2026. https://ginasthma.org/wp-content/uploads/2024/05/GINA-2024-Strategy-Report-24_05_22_WMS.pdf

