# Supplementary material for: Airway inflammation and asthma control in patients using salbutamol or budesonide/formoterol reliever: a randomised, controlled study
Source: ERJ Open Res. 2026 Sep 28;12(5):00116-2026. doi: 10.1183/23120541.00116-2026 (PMC13617593; doi:10.1183/23120541.00116-2026)
Supplement: Supplementary file 1 [file 00116-2026.SUPPLEMENT.pdf]

Supplementary Materials

Figure S1. Variation in evening PEF a) and evening FEV_1 b) in patients receiving BUD/FORM MART or BUD/FORM maintenance therapy plus SABA during the 24-week treatment period (full analysis set*)

a) Evening PEF **b) Evening FEV_1**

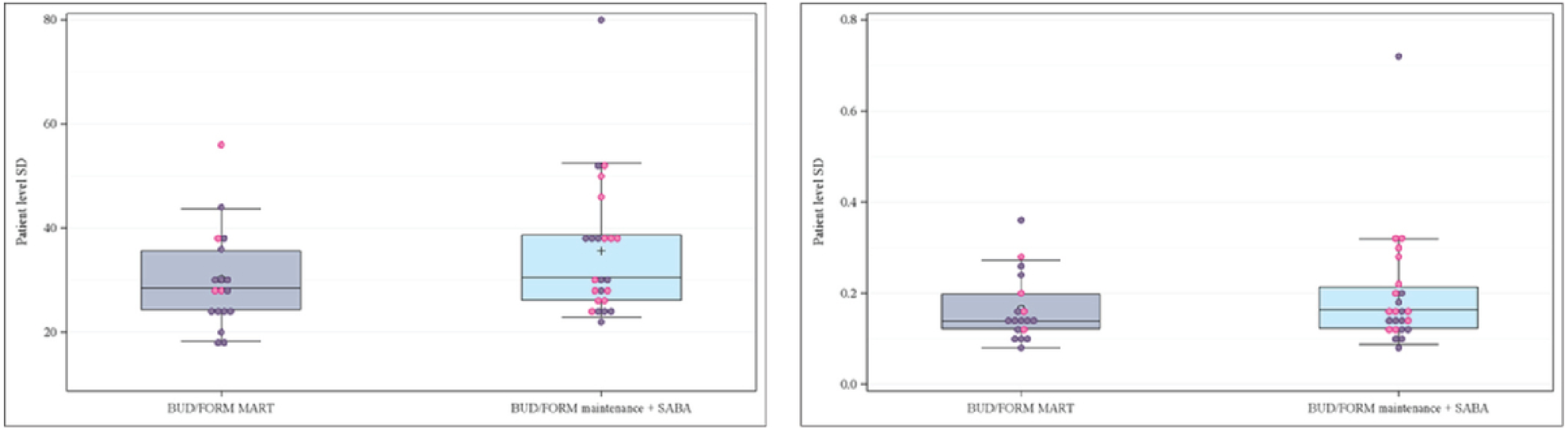

Dots show SD values for a given parameter during the 24-week treatment period for individual patients; pink dots represent patients who experienced a clinical event (≥1 SevEx and/or CompEx) and purple dots represent patients without a clinical event (without ≥1 SevEx and/or CompEx). Box shows the IQR, spanning from Q1 to Q3, representing the middle 50% of the data. The horizontal line inside the box represents the median. Whiskers extending from the box represent the minimum and maximum non-outlier values (smallest and largest values within 1.5 x IQR from the quartiles). Individual points plotted outside of the whiskers are "outliers" (values that are more than 1.5 x IQR from the quartiles). The diamond inside the box represents the mean.

*All randomised patients with ≥1 post baseline measurement. Summary statistics are based on individual patient-level SD to summarise variation over the observational period by treatment arm.

BUD: budesonide; CompEx: composite surrogate endpoint for severe exacerbations of asthma; FeNO: fractional exhaled nitric oxide; FEV_1: forced expiratory volume in one second; FORM: formoterol; IQR: interquartile range; MART: maintenance and reliever therapy; PEF: peak expiratory flow; Q1: first quartile; Q3: third quartile; SABA: short-acting β_2-agonist; SD: standard deviation; SevEx: severe exacerbation.

Figure S2. Patient-level variation in evening PEF a), and evening FEV_1 b) in adults with asthma receiving BUD/FORM MART or BUD/FORM maintenance therapy plus SABA during the 24-week treatment period (full analysis set)

a) Evening PEF

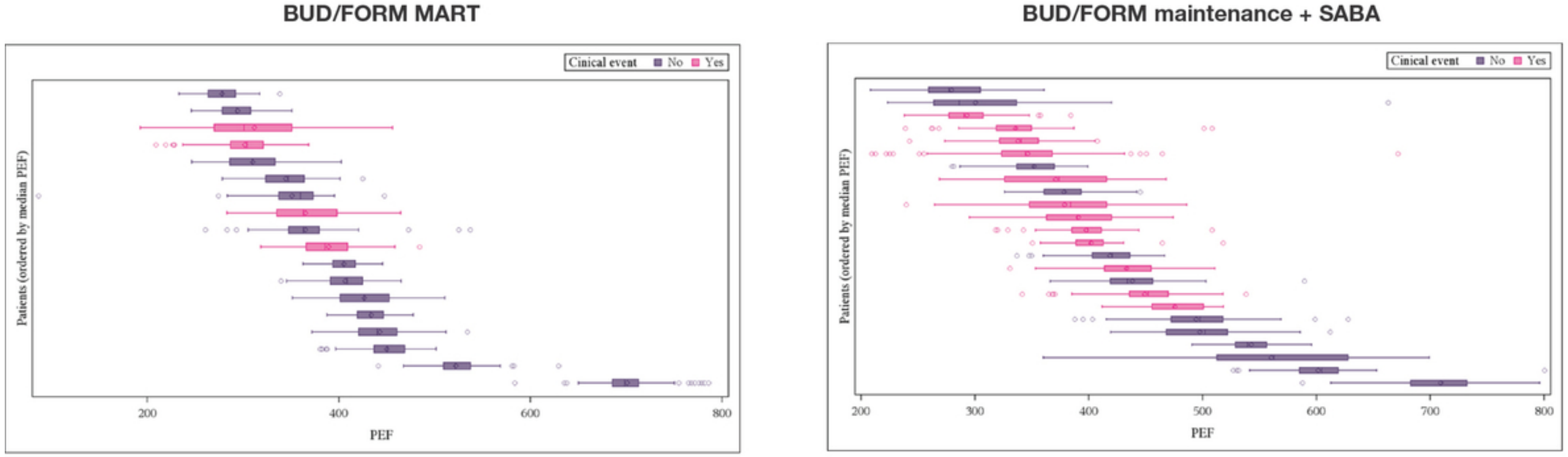

b) Evening FEV_1

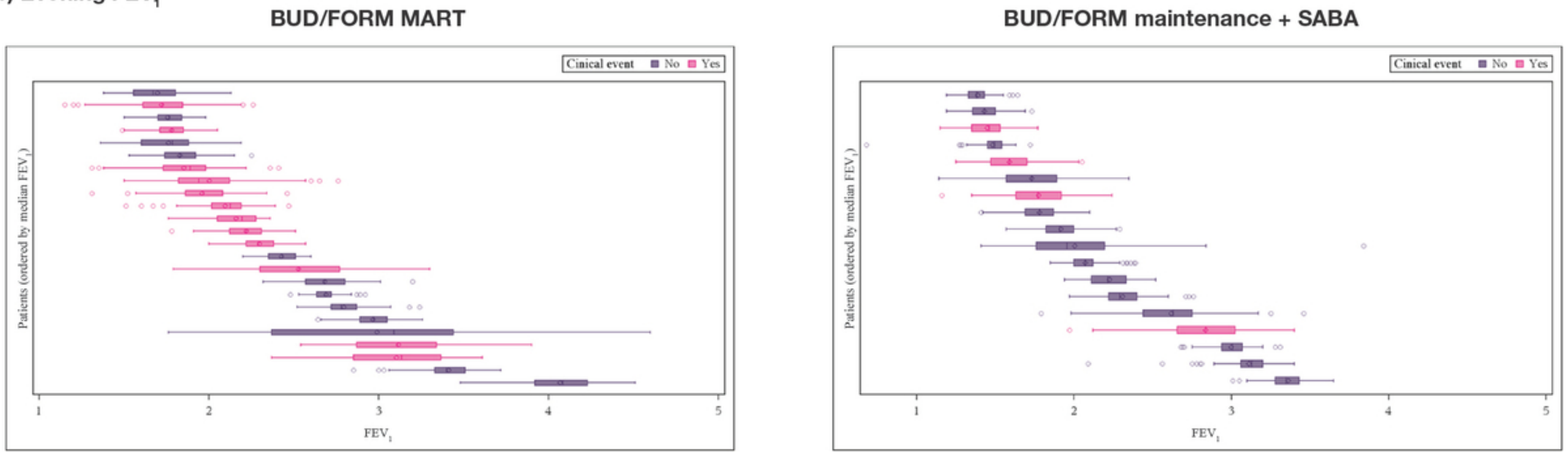

Each line represents an individual patient; pink denotes those with a clinical event (≥1 SevEx and/or CompEx); purple denotes those without a clinical event (without ≥1 SevEx and/or CompEx). Box shows the IQR, spanning from Q1 to Q3, representing the middle 50% of the data. The horizontal line inside the box that marks the median. Whiskers extending from the box represent the minimum and maximum non-outlier values (smallest and largest values within 1.5 x IQR from the quartiles). Individual points plotted outside of the whiskers are "outliers" (values that are more than 1.5 x IQR from the quartiles). The diamond inside the box represents the mean.

BUD: budesonide; CompEx: composite surrogate endpoint for severe exacerbations of asthma; FeNO: fractional exhaled nitric oxide; FEV_1: forced expiratory volume in one second; FORM: formoterol; IQR: interquartile range; MART: maintenance and reliever therapy; PEF: peak expiratory flow; Q1: first quartile; Q3: third quartile; SABA: short-acting β_2-agonist; SevEx: severe exacerbation.
